# Re-localization of Cellular Protein SRp20 during Poliovirus Infection: Bridging a Viral IRES to the Host Cell Translation Apparatus

**DOI:** 10.1371/journal.ppat.1002127

**Published:** 2011-07-14

**Authors:** Kerry D. Fitzgerald, Bert L. Semler

**Affiliations:** Department of Microbiology and Molecular Genetics, School of Medicine, University of California, Irvine, California, United States of America; Columbia University, United States of America

## Abstract

Poliovirus IRES-mediated translation requires the functions of certain canonical as well as non-canonical factors for the recruitment of ribosomes to the viral RNA. The interaction of cellular proteins PCBP2 and SRp20 in extracts from poliovirus-infected cells has been previously described, and these two proteins were shown to function synergistically in viral translation. To further define the mechanism of ribosome recruitment for the initiation of poliovirus IRES-dependent translation, we focused on the role of the interaction between cellular proteins PCBP2 and SRp20. Work described here demonstrates that SRp20 dramatically re-localizes from the nucleus to the cytoplasm of poliovirus-infected neuroblastoma cells during the course of infection. Importantly, SRp20 partially co-localizes with PCBP2 in the cytoplasm of infected cells, corroborating our previous *in vitro* interaction data. In addition, the data presented implicate the presence of these two proteins in viral translation initiation complexes. We show that in extracts from poliovirus-infected cells, SRp20 is associated with PCBP2 bound to poliovirus RNA, indicating that this interaction occurs on the viral RNA. Finally, we generated a mutated version of SRp20 lacking the RNA recognition motif (SRp20ΔRRM) and found that this protein is localized similar to the full length SRp20, and also partially co-localizes with PCBP2 during poliovirus infection. Expression of this mutated version of SRp20 results in a ∼100 fold decrease in virus yield for poliovirus when compared to expression of wild type SRp20, possibly via a dominant negative effect. Taken together, these results are consistent with a model in which SRp20 interacts with PCBP2 bound to the viral RNA, and this interaction functions to recruit ribosomes to the viral RNA in a direct or indirect manner, with the participation of additional protein-protein or protein-RNA interactions.

## Author Summary

Picornaviruses are positive-sense RNA viruses that cause diseases ranging from the common cold to poliomyelitis. Poliovirus is one of the most extensively studied members of the *Picornaviridae* family. However, a complete understanding of the mechanism by which the viral RNA genome directs the synthesis of its protein products is lacking. Poliovirus usurps the host cell translation machinery to initiate viral polyprotein synthesis via a mechanism distinct from the cellular cap-binding, ribosome scanning model of translation. This allows the virus to down-regulate host cell translation while providing an advantage for its own gene expression. Owing to its small genome size, poliovirus utilizes host cell proteins to facilitate the recruitment of the translation machinery, a process that is still not completely defined. Previous work highlighted the importance of two particular host cell RNA binding proteins in poliovirus translation. Here we employ imaging techniques, fractionation assays, and RNA binding experiments to further examine the specific role these proteins play in poliovirus translation. We also generated a truncated version of one of the proteins and observed a dramatic effect on virus growth, highlighting its significance during poliovirus infection and supporting our model for bridging the cellular translation apparatus to viral RNA.

## Introduction

**Figure 1:**
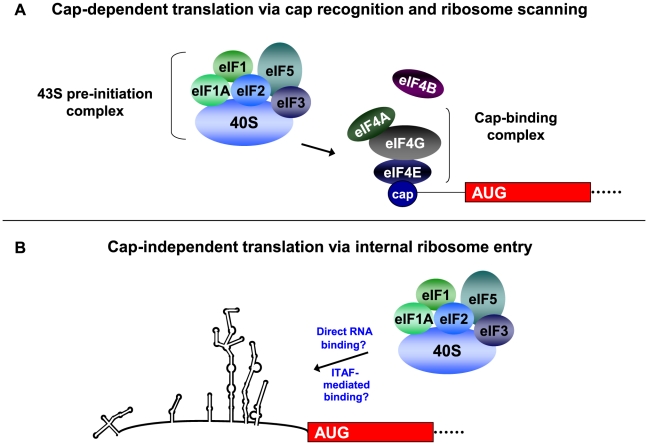
Recruitment of the 43S pre-initiation complex for cap-dependent and cap-independent translation initiation. For cap-dependent translation initiation (Figure 1A), the eIF4F cap-binding complex recognizes and binds to the 5′ cap structure of the mRNA. Following cap binding and ribosome scanning mediated by the 43S pre-initiation complex, initiation occurs at an AUG in a favorable context. After GTP hydrolysis and 60S subunit joining, the ribosome is elongation-competent and protein synthesis begins. In the cap-independent mechanism of translation initiation (Figure 1B), the 43S pre-initiation complex associates with RNA sequences in the IRES either directly or in conjunction with canonical or non-canonical initiation factors, which facilitates initiation at the appropriate AUG start codon. Non-canonical factors are indicated as IRES trans-acting factors, or ITAFs. This figure highlights the major differences in the mechanisms of cap-dependent and cap-independent translation, and is not a comprehensive model for eukaryotic translation initiation. Additional canonical factors (such as eIF4G and eIF4B) as well as non-canonical factors have been shown to bind to the poliovirus IRES and/or stimulate poliovirus translation. (Figure taken from [2], with permission).

Translation of eukaryotic mRNAs most often occurs via a cap-dependent mechanism of initiation (see Figure 1A). Cellular mRNAs contain a 7-methyl guanosine cap at their 5′ ends, and this cap structure is recognized by the eukaryotic initiation factor 4F (eIF4F) cap binding complex. The eIF4F complex consists of the initiation factors 4A, 4G, and 4E and recruits the ribosome to the mRNA for translation initiation. The 40S ribosomal subunit binds a protein complex that consists of eIF1, eIF2-GTP-Met-tRNA (i.e., the ternary complex), eIF3, and eIF5. The assembled 43S pre-initiation complex binds the mRNA at the cap structure via interaction of a central domain of eIF4G with eIF3. The bound pre-initiation complex scans along the RNA until an AUG start codon is recognized in a favorable context [1], at which point GTP is hydrolyzed to GDP in the presence of eIF5. Large ribosomal subunit joining then occurs to generate an elongation-competent 80S ribosome and protein synthesis begins; initiation factors are recycled for subsequent rounds of initiation. The cellular protein poly(A)-binding protein (PABP), which binds the 3′ poly(A) tracts of cellular mRNAs and interacts with eIF4G, allows for circularization of the mRNA and provides a context for multiple rounds of translation initiation. Changes to the cellular environment, which can occur during viral infection or under various conditions of stress, can result in a down-regulation of cap-dependent translation often by interfering with initiation factors that play important roles in cap-dependent translation initiation.

Unlike cap-dependent translation, the mechanism of cap-independent ribosome recruitment to the RNA has not been fully defined (see Figure 1B). The 40S ribosomal subunit recognizes an RNA sequence, structure, or ribonucleoprotein complex within the 5′ noncoding region (5′ NCR) of the RNA, and translation initiation can occur several hundred nucleotides downstream from the 5′ end of the RNA. A 5′ cap is not required for assembly of initiation factors for this alternative form of initiation, so cap-recognition of the 40S ribosomal subunit via the intact eIF4F cap binding complex does not occur. In addition, the RNA is generally highly structured in nature, and ribosomes may not be capable of scanning through the noncoding region to reach the authentic initiation site. Therefore, initiation involves the internal binding of ribosomes to the RNA. Thus, cap-independent translation involves features that are distinct from the canonical cap-binding, ribosome scanning model, and these factors highlight important differences between cap-dependent and cap-independent translation initiation.

Internal entry of ribosomes for translation initiation was first observed for picornavirus RNAs, and has since been identified in other viruses as well as a subset of cellular mRNAs (for review, see [2], [3], [4]). The poliovirus and encephalomyocarditis virus (EMCV) viral genomic RNAs were the first RNAs found to contain sequences in their 5′ NCRs, termed internal ribosome entry sites (IRESes), which mediate the internal binding of ribosomes for translation initiation [5], [6], [7]. During almost all picornavirus infections, cap-dependent translation is shut-down via cleavage of translation factors by viral proteinases. The viruses utilize IRES-mediated translation initiation to direct the synthesis of viral proteins.

Picornaviruses are cytoplasmic RNA viruses containing a ∼7.0 kb–8.5 kb positive-sense single-stranded genome. This genomic RNA encodes a single open-reading frame, which is translated to generate a polyprotein that is proteolytically processed. Viral proteinases 2A and 3C cleave several cellular proteins, including eIF4G, to down-regulate host cell translation during poliovirus, human rhinovirus, or coxsackievirus infection. Other work has shown that poliovirus and coxsackievirus proteinases cleave PABP and that this cleavage correlates with host cell translation shut-off [8], [9], [10], [11], [12], [13]. The virus can then utilize available translation machinery and host cell proteins to direct IRES-mediated translation of viral proteins and no longer has to compete with cellular mRNA translation for these factors.

Owing to their limited coding capacity, picornaviruses have evolved to utilize certain host cell proteins along with encoded viral proteins and RNA secondary structures to direct viral translation and RNA replication. Certain IRES trans-activating factors (ITAFs) are known to bind to the 5′ NCRs of picornavirus RNAs, and a subset of these has been shown to have an effect on viral translation (for review, see [2], [4]). Poly(rC) binding protein 2, or PCBP2, is one ITAF that has been extensively studied for its role in picornavirus translation. PCBP2 is a 38 kDa cellular RNA binding protein that has been associated with RNA stability and translational regulation of cellular mRNAs [14]. This protein is also known to bind the 5′ NCR of several picornavirus RNAs [15]. For poliovirus, the binding of PCBP2 to stem-loop IV in the IRES is required for translation, and depletion of PCBP2 from cell-free extracts results in a dramatic decrease in viral translation [16], [17]. Poliovirus also requires the interaction of PCBP2 with cellular splicing factor SRp20, and these two proteins have been shown to function synergistically in poliovirus translation [18]. The specific role this interaction plays in viral translation is not yet completely understood.

SR proteins are a family of splicing factors that function in constitutive splicing as well as in alternative splice site selection in the nucleus of eukaryotic cells [19]. Members of the SR family of proteins contain one or more RNA-recognition motifs (RRMs) at their N-termini and a serine/arginine-rich (RS) domain at their C-termini. The RRM domains are involved in RNA binding, and the RS domain has been implicated in protein shuttling and protein-protein interactions [20], [21]. SRp20 is a 20 kDa RNA binding protein that shuttles between the nucleus and cytoplasm of cells, and contains one RRM and one RS domain. SR proteins have been extensively studied, and much of the early work focused on mRNA splicing and stability. Beyond their resident functions in the nucleus, several SR proteins (including SRp20) have also been implicated in mRNA nuclear export in eukaryotic cells, and provide a possible connection between splicing, export, and translation ([22]; for review, see [23], [24]). More recently it was proposed that the SR protein ASF/SF2 functions in cap-dependent translation, and that SR protein 9G8 functions in the translation of unspliced mRNAs containing a constitutive transport element [24], [25], [26]. In addition, SRp40 and SRp55 were found to promote HIV-1 Gag translation from unspliced, intron-containing viral RNAs; thus, it was proposed that SR proteins couple HIV-1 genomic RNA biogenesis to translation functions [27]. Taken together, these recent studies suggest a role for SR proteins in linking nuclear splicing and export with translation in eukaryotic cells, and provide evidence for the role of SR proteins in both cap-dependent translation and translation of certain viral RNAs.

In this study we sought to further define the mechanism of ribosome recruitment to poliovirus RNA, focusing specifically on the role of the interaction between PCBP2 and SRp20. We observed a dramatic re-localization of SRp20 from the nucleus to the cytoplasm of poliovirus-infected cells, where we also observed partial co-localization with PCBP2 during infection. Using sucrose gradient sedimentation of extracts from poliovirus-infected cells, we demonstrated that both PCBP2 and SRp20 partially co-sediment in translation initiation complex-containing fractions. We also determined that SRp20 is associated with PCBP2 bound to poliovirus stem-loop IV. Finally, we designed a deleted version of SRp20 lacking one of its functional domains (denoted SRp20ΔRRM), and determined that its localization in mock- and poliovirus-infected cells (and co-localization with PCBP2) resembled that of the wild type SRp20 protein. Significantly, expression of SRp20 lacking the RRM domain resulted in an approximate two-log decrease in virus yield for poliovirus when compared to expression of wild type SRp20. Taken together, these results support the model that SRp20 interacts with PCBP2 on poliovirus RNA, and this interaction functions either directly or indirectly to recruit the translation machinery to the viral RNA for IRES-mediated translation initiation.

## Materials and Methods

### Cell culture and DNA constructs

HeLa cells [28], [29] were grown in suspension culture or as monolayers in Dulbecco's Modified Eagle's Media (DMEM) supplemented with 8% newborn calf serum (NCS). SK-N-SH (American Type Culture Collection number: HTB-11) cells were grown in DMEM supplemented with 20% fetal calf serum (FCS). For imaging experiments, pEGFP and pEGFP-SRp20 plasmids (containing CMV promoters for expression) were kindly provided by Dr. Roz Sandri-Goldin. To generate the SRp20 deletion mutant SRp20ΔRRM, an EcoRI site was engineered at the beginning of the coding sequence for the RS domain (preceding the TAP-binding region, [30]). Following digestion to remove the RRM fragment that was produced, the linear vector was gel-purified, phenol-chloroform extracted and ethanol precipitated, and then re-ligated with T4 DNA ligase (New England BioLabs) to re-circularize the plasmid. Removal of the RRM was verified via sequencing. For DNA co-transfection experiments, poliovirus pPVA55 cDNA [31] was utilized. For RNA affinity assays, stem-loop IV RNA was generated from poliovirus subclone cDNA p220–460 [32].

### Laser Scanning Confocal Microscopy (LSCM) experiments

SK-N-SH cells or HeLa cells seeded on coverslips in a six-well plate format, and allowed to grow to approximately 70% confluency. Cells were transfected with pEGFP, pEGFP-SRp20, or pEGFP-SRp20ΔRRM using Fugene transfection reagent (Roche). Twenty-four hours post-transfection, cells were infected with poliovirus [multiplicity of infection (MOI) = 25], and at specific times post-infection cells were washed with phosphate-buffered saline (PBS) and fixed with 3.7% paraformaldehyde at room temperature for 20 min. Cells were washed with PBS twice, and cell membranes were permeabilized with 0.5% NP-40 in PBS for 5 min. Cells were washed with 1% NCS in PBS and incubated with normal donkey serum (Jackson ImmunoResearch) for one hour to block nonspecific interactions. Cells were then incubated with a monoclonal antibody against PCBP2 [33] for one hour. Cells were washed and incubated with a goat-anti mouse secondary antibody conjugated with biotin (Pierce) for 30 min, and then incubated with streptavidin conjugated with Texas Red (GE Healthcare) for 30 min. Cells were washed with 1% NCS-PBS and incubated with DAPI to stain nuclei. Cells were washed with PBS, and coverslips were mounted on microscope slides with mounting media (Biomeda) and allowed to dry overnight at room temperature. Coverslips were sealed with nail polish, and cells were imaged using a Zeiss LSM 510 multi-photon laser scanning confocal microscope. Images were processed using the LSM 510 software.

### Generation of cytoplasmic extracts from mock- and poliovirus-infected cells

HeLa cells were grown in suspension culture to approximately 6×10^5^ cells/ml. Cells were pelleted and resuspended in DMEM to a concentration of approximately 5×10^6^ cells/ml. Cells were mock-infected or infected with poliovirus (MOI = 25) in spinner flasks. At each hour post-infection an aliquot of cells was withdrawn from each flask, incubated with cycloheximide for 5 min, and then pelleted. Cell pellets were washed twice with ice-cold PBS and resuspended in 0.3% NP-40 lysis buffer (20 mM Tris pH 7.5, 5 mM MgCl_2_, 100 mM KCl, 0.3% NP-40, 0.1 mg/ml cycloheximide). Cells were lysed for 20 min on ice, and lysates were cleared by centrifugation at 5,000× g for 10 min at 4°C. Lysates were aliquoted and stored at −70°C until use.

### Sucrose gradient fractionation of cytoplasmic extracts

A two-chamber mixer was used to generate 7%–47% (w/w) sucrose gradients (comprised of 20 mM Tris pH 7.5, 5 mM MgCl_2_, and 100 mM KCl). An aliquot of extract from either mock- or poliovirus-infected cells was thawed and carefully overlaid onto the top of the gradient. Gradients were centrifuged for two hours at 35,000 RPM at 4°C in a Beckman SW41 swinging bucket rotor. Following centrifugation, gradients were fractionated using an Isco fractionator by piercing at the bottom of the tube and chasing the gradient with a 60% sucrose solution. Fractions were collected with concomitant measurement of the OD 254 nm and were stored at −70°C until further processing. For Western blot analysis, antibodies were used to detect the sedimentation of ribosomal subunits and assembled ribosomes (anti-S6, Cell Signaling; anti-P0, ImmunoVision). Antibodies against eIF2α (Cell Signaling) and PABP (a gift from Dr. Richard Lloyd) were used to detect the sedimentation of these proteins. PCBP2 antibody was previously described [33]; an antibody against SRp20 was obtained from Invitrogen.

### RNA affinity assays

Plasmid subclone DNA p220–460 [32] was linearized with HindIII (New England BioLabs), phenol-chloroform extracted, and ethanol precipitated. Transcript RNA was produced from linear DNA with the T7 MegashortScript Kit (Ambion) using a 4∶1 ratio of Biotin-CTP (Invitrogen) to CTP. Biotinylated transcript RNA was purified using an RNeasy column (Qiagen). Interaction of the biotinylated RNA with streptavidin protein was verified using agarose gel shift analysis (data not shown). For RNA affinity experiments, 500 pmol of biotinylated stem-loop IV RNA was used per reaction and tRNA (25 µg/ml) was included as a control for nonspecific interactions. Biotinylated stem-loop IV RNA was incubated with streptavidin agarose (Sigma) for one hour on ice with occasional vortexing to allow for association of the RNA with the matrix. The agarose was washed twice in 400 µl of 50 mM KCl buffer (50 mM KCl, 5% glycerol, 1 mM DTT, 0.5 mM EDTA pH 8, 2.5 mM MgCl_2_, 25 µg/ml tRNA) and then resuspended in 400 µl of 50 mM KCl buffer. Extracts described previously for sucrose gradient fractionation (from poliovirus-infected cells at three or four hours post-infection) were utilized as a source of PCBP2 and SRp20. 400 µg of extract was initially pre-cleared by incubating the extract with streptavidin agarose on ice for one hour. The agarose was pelleted and removed, and pre-cleared extract was incubated with biotinylated stem-loop IV-streptavidin agarose for two hours on ice, vortexing occasionally. Bound complexes were washed three times with 100 mM KCl buffer (same as 50 mM KCl buffer except for the higher concentration of KCl) and resuspended in 50 µl 2× Laemmli sample buffer. Samples were boiled and subjected to SDS-PAGE and Western blot analysis. When poly(rC) was included as a competitor for PCBP2 binding, 1 nmol synthetic poly(rC) RNA (Thermo Scientific) was pre-incubated with the pre-cleared extract for 1 hour on ice prior to incubation with the biotinylated poliovirus stem-loop IV-streptavidin agarose.

### Poliovirus growth assays

HeLa cells were plated in 60 mm dishes and grown to approximately 70% confluency. Cells were co-transfected with poliovirus cDNA plasmid pPVA55 and either pEGFP, pEGFP-SRp20, or pEGFP-SRp20ΔRRM using Fugene transfection reagent (Roche). Ninety-six hours post-transfection, cells and media were harvested via scraping. Virus was released by five cycles of freeze-thaw, and virus samples were serially-diluted. Dilutions were used to infect HeLa cells (80–90% confluent) in 60 mm dishes; following a 30 min adsorption period at room temperature, a 0.45% agarose-DMEM overlay was added to the cells, and plaques were allowed to develop. After 24 hours, another layer of 0.45% agarose-DMEM was added to the cells. After 48 hours, cells were treated with 10% TCA, and plaques were stained with crystal violet and counted. All experiments were carried out in triplicate.

## Results

### SRp20 displays a dramatic re-localization from the nucleus to the cytoplasm of the cell during poliovirus infection

**Figure 2:**
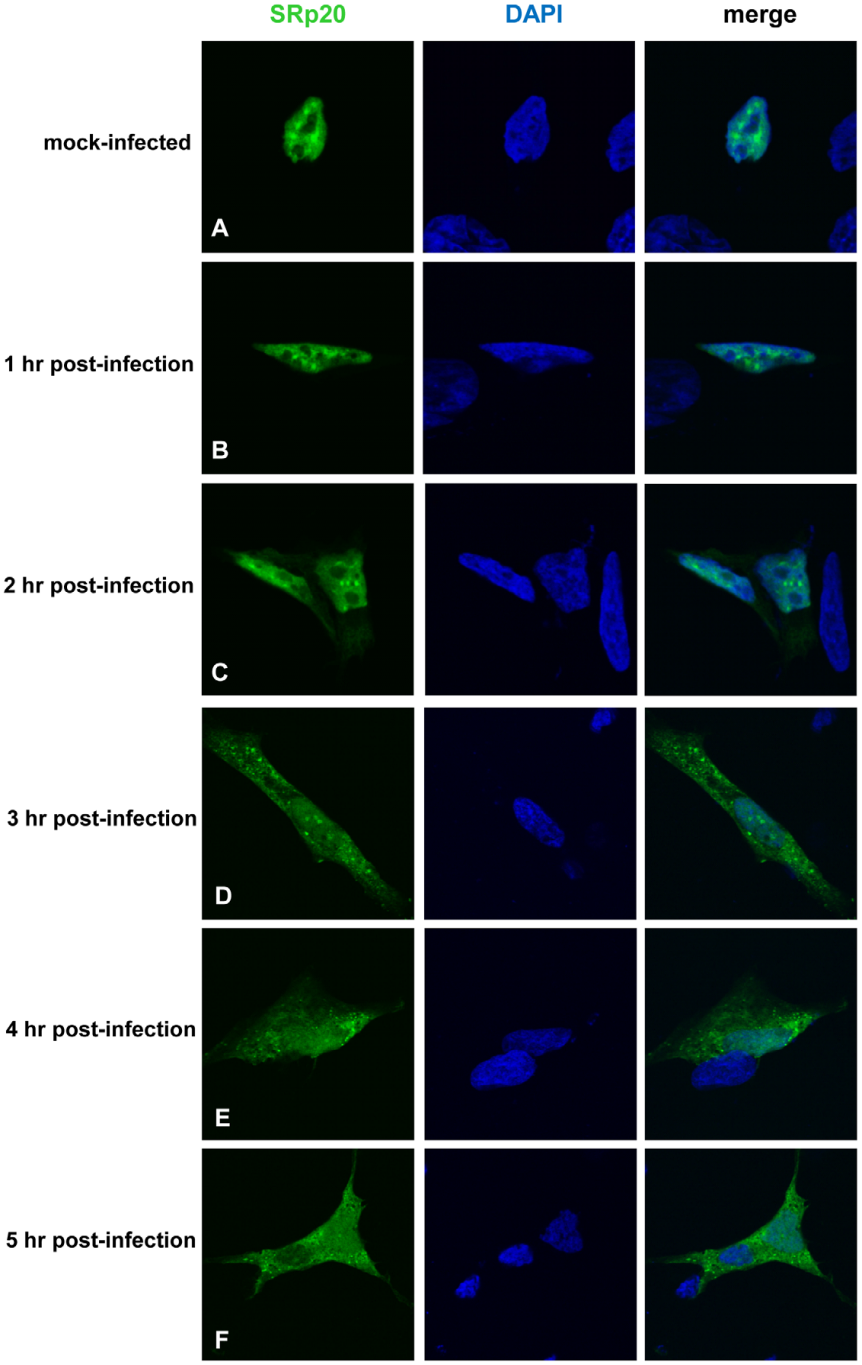
SRp20 re-localization from the nucleus to the cytoplasm of SK-N-SH cells during poliovirus infection. Cells were transfected with GFP-SRp20 and either mock-infected (A) or infected with poliovirus at an MOI of 25. Cells were fixed at specific times post-infection (1–5 hours, B–F) and imaged. SRp20 localization was determined using confocal microscopy; nuclei were identified by DAPI staining.

**Figure 3:**
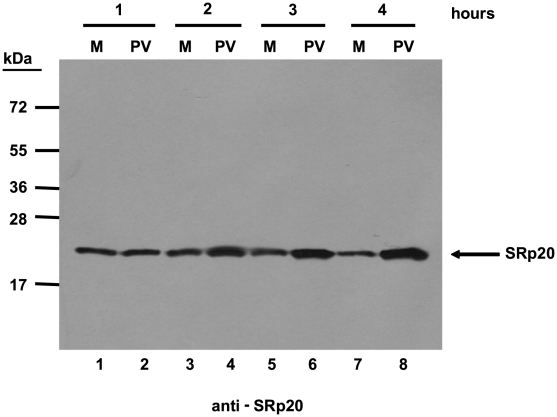
SRp20 is present in cytoplasmic extracts from both mock- or poliovirus-infected cells. Cytoplasmic extracts from mock- or poliovirus-infected cells (hours 1 through 4 as indicated) generated for sucrose gradient fractionation were also examined for the presence of SRp20. Extracts (100 µg of total protein) were subjected to SDS-PAGE and Western blot analysis, probing with an SRp20 monoclonal antibody. Lanes marked ‘M’ are extracts from mock-infected cells; lanes marked ‘PV’ are extracts from poliovirus-infected cells.

We first determined the subcellular localization of SRp20 during poliovirus infection. To visualize SRp20 in the cell, we transfected a plasmid encoding a GFP-tagged version of the protein into SK-N-SH cells, a neuroblastoma cell line permissive for poliovirus infection. We subsequently utilized confocal microscopy to capture fixed images of SRp20 localization in mock- and poliovirus-infected cells over a time course. The results of these experiments are shown in Figure 2. SRp20 is a nuclear splicing factor, therefore we expected this protein to be localized predominantly in the nucleus in cells under normal conditions, with a portion of the protein shuttling between the nucleus and the cytoplasm. Since SRp20 is important for poliovirus infection, which occurs in the cytoplasm of the cell, we predicted that we would observe a greater amount of SRp20 in the cytoplasm of poliovirus-infected cells compared to mock-infected cells. We determined that SRp20 is indeed predominantly localized in the nucleus in mock-infected cells (see Figure 2A), and this localization in mock-infected cells did not change over the time course that was carried out (data not shown). While a portion of the SRp20 shuttles between the nucleus and the cytoplasm, the prominent signal in the nucleus precludes visualizing the small portion of SRp20 in the cytoplasm at any given time (which is consistent with published data for this protein, [34]). At 1 hour post-infection SRp20 remained predominantly nuclear in localization (Figure 2B). However, at 2 hours post-infection SRp20 could be visualized re-localizing to some extent to the cytoplasm of the infected cell (Figure 2C). At 3 hours post-infection, a more dramatic re-localization of SRp20 into the cytoplasm of the infected cell was observed (Figure 2D). Later in infection (4 and 5 hours post-infection, Figure 2E and 2F), much of the SRp20 was localized in the cytoplasm of the cell with little of the protein remaining in the nucleus. The presence of cytoplasmic SRp20 was also confirmed via nuclear-cytoplasmic fractionation and Western blot analysis (Figure 3). Equal total protein amounts of extracts from both mock- and poliovirus-infected cells (1 through 4 hours) were subjected to SDS-PAGE and examined via Western blot, probing with an SRp20 monoclonal antibody. SRp20 was found in both types of cytoplasmic extracts at all times examined. The increased accumulation of SRp20 in the cytoplasm of infected cells could also be observed in the Western blot, beginning about 2 hours post-infection (compare lanes 3 and 4) and it continued to increase over the course of infection (compare lanes 5 and 6, and lanes 7 and 8). Thus, SRp20 re-localizes to a large extent from the nucleus to the cytoplasm of cells during poliovirus infection, and can be visualized re-localizing to the cytoplasm beginning about 2 hours post-infection.

**Figure 4:**
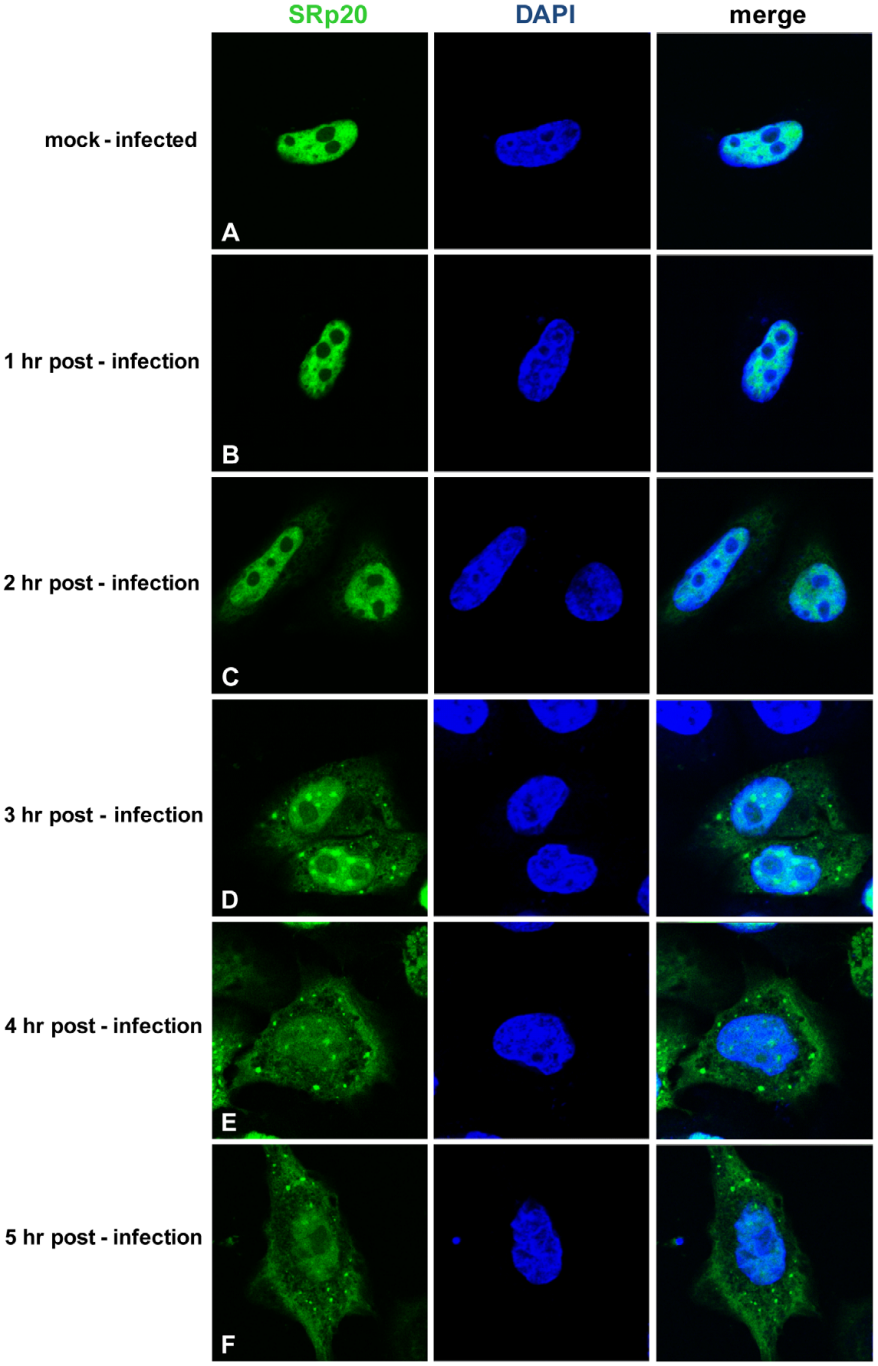
SRp20 re-localization from the nucleus to the cytoplasm of HeLa cells during poliovirus infection. Cells were transfected with GFP-SRp20 and either mock-infected (A) or infected with poliovirus at an MOI of 25. Cells were fixed at specific times post-infection (1–5 hours, B–F) and imaged. SRp20 localization was determined using confocal microscopy; nuclei were identified by DAPI staining.

Other work has examined the nucleo-cytoplasmic trafficking of proteins during poliovirus infection in HeLa cells (for example, [35]); in addition, a portion of our studies presented here include biochemical work utilizing these cells. Therefore, we also investigated SRp20 localization during poliovirus infection in HeLa cells (Figure 4). Similar to what is seen in SK-N-SH cells, SRp20 is predominantly nuclear in mock-infected cells (Figure 4A), and remains mostly nuclear at 1 hour post-infection (Figure 4B). At 2 hours post-infection, SRp20 begins to re-localize to the cytoplasm of the infected cell (Figure 4C), which becomes very apparent at 3 hours post-infection (Figure 4D). Figure 4E and 4F show the dramatic re-localization of SRp20 at later times post-infection with poliovirus. Additional experiments were also carried out using a monoclonal antibody to label endogenous SRp20, and we determined that the endogenous protein re-localizes from the nucleus to the cytoplasm during poliovirus infection (data not shown). Transfection of the GFP-tagged version of the protein provided the advantage of a consistent signal from the GFP fluorescence, in contrast to labeling SRp20 with the monoclonal antibody, which resulted in a higher variability of the signal produced. GFP-SRp20 has been previously characterized to localize and function similarly to endogenous SRp20 [36]. We also determined that the GFP tag alone does not contribute to the localization of the protein (data not shown).

### SRp20 partially co-localizes with PCBP2 in the cytoplasm of poliovirus-infected cells

**Figure 5:**
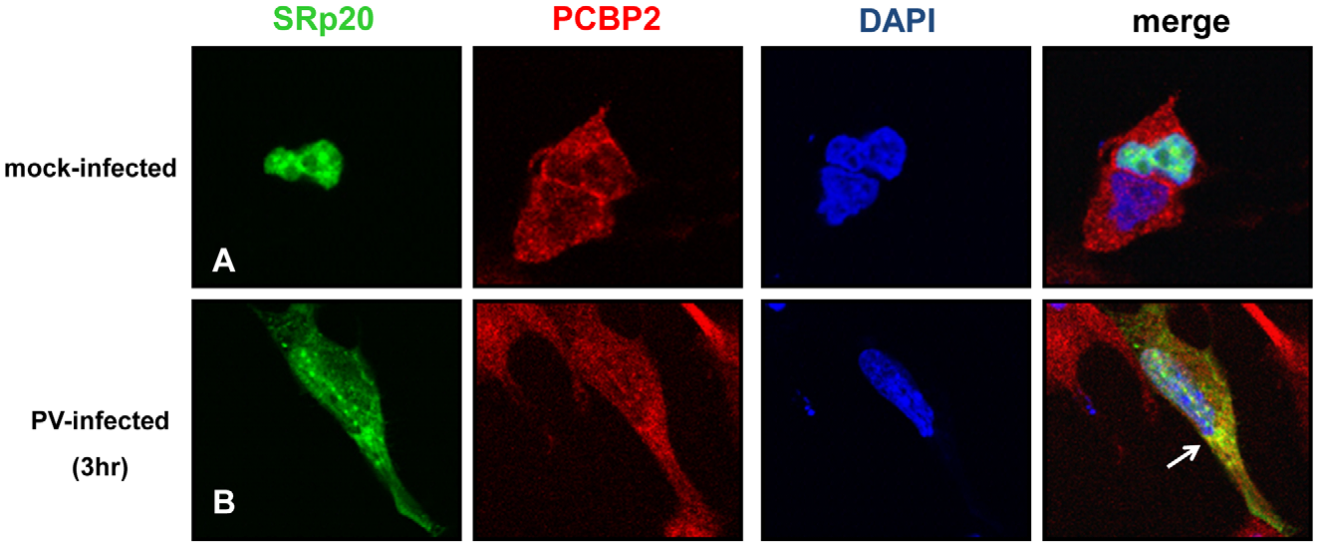
SRp20 partial co-localization with PCBP2 in the cytoplasm of poliovirus-infected SK-N-SH cells. Cells were transfected with GFP-SRp20 and mock-infected (A) or infected with poliovirus for 3 hours (B) at an MOI of 25. At 3 hours post-infection, cells were fixed and incubated with a PCBP2 monoclonal antibody, a secondary antibody conjugated to biotin, and streptavidin conjugated to Texas Red. Cells were examined for co-localization of PCBP2 and SRp20 (shown in the merged image in yellow and highlighted by the white arrow) in the cytoplasm of the cells using confocal microscopy; nuclei were identified by DAPI staining.

It has been previously shown that SRp20 interacts with PCBP2, and this interaction is required for poliovirus IRES-mediated translation [18]. These studies utilized *in vitro* assays such as co-immunoprecipitations using extracts from poliovirus-infected HeLa cells, and GST pull down assays using recombinant PCBP2 and SRp20. To further study this interaction in intact cells during poliovirus infection, we transfected neuroblastoma cells (SK-N-SH) with the GFP-SRp20 plasmid and subsequently infected with poliovirus. Cells were fixed at 3 hours post-infection and then labeled with a PCBP2 antibody. To determine the subcellular location of these two proteins and their predicted close association with each other, we again used confocal microscopy to visualize potential co-localization of SRp20 and PCBP2. The results of these experiments are shown in Figure 5. We took advantage of the SK-N-SH cell line for these co-localization experiments because we expected that co-localization of PCBP2 and SRp20 would occur in the cytoplasm of the cell, and SK-N-SH cells have a more distinct cytoplasmic space compared to other cell types, such as HeLa cells. We examined cells at 3 hours post-infection because sufficient amounts of SRp20 would be expected to be re-localized into the cytoplasm of the cell, where potential co-localization with PCBP2 could be visualized. In the mock-infected cells, SRp20 is predominantly nuclear in localization (as observed previously in Figure 2) while PCBP2 was found to be localized in both the nucleus and the cytoplasm (consistent with published results for this protein [37], [38], see Figure 5A). At 3 hours post-infection SRp20 re-localized to the cytoplasm of the infected cell and partially co-localized with PCBP2 (shown in Figure 5B). Neither PCBP2 nor SRp20 occupied all of the cytoplasmic space in the infected cells, as regions could be observed to contain one protein or the other, but not both. The co-localization observed was in the cytoplasm of the infected cell and was distinct from the nucleus; this was confirmed via z-stack analysis (data not shown). The z-stacks of some images were further processed using the Volocity Image Analysis Software (PerkinElmer) to generate rotational movies of the cells in three-dimensions (see Video S1). Thus, SRp20 and PCBP2 can be visualized in very close proximity to each other in the cytoplasm of intact cells during poliovirus infection.

### SRp20 and PCBP2 partially sediment in translation initiation complex-containing fractions generated using extracts from mock- or poliovirus-infected cells

**Figure 6:**
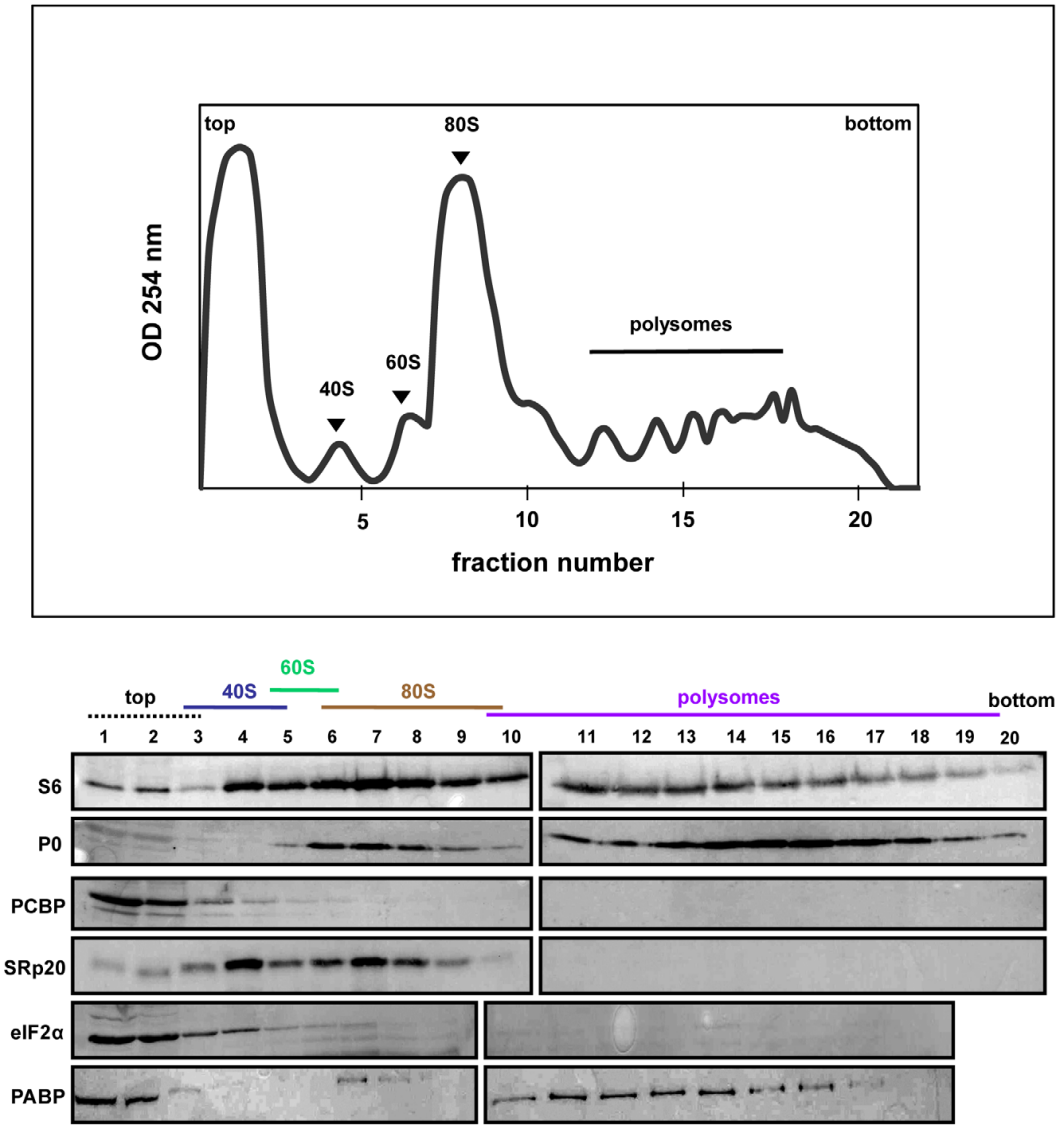
SRp20 and PCBP2 co-sedimentation with 40S ribosomal subunits in mock-infected cells. Extracts were generated from mock-infected HeLa cells, sedimented through 7%–47% sucrose gradients and fractionated. The collected fractions were subjected to Western blot analysis to determine the co-sedimentation of PCBP2 and SRp20 with ribosomal subunits, monosomes, or polysomes. Representative polysome profiles and Western blot analyses are shown.

SRp20 and PCBP2 are both important for poliovirus IRES-mediated translation, as depletion of one or both proteins reduces levels of viral translation. To investigate whether SRp20 and PCBP2 are associated with translation initiation complexes and/or actively translating polysomes, we carried out sucrose gradient fractionation of extracts from mock- or poliovirus-infected HeLa cells. Fractionation of extracts generated polysome profiles, which were used to determine the sedimentation of ribosomal subunits (40S and 60S peaks), assembled monosomes (80S peak), and actively translating polysomes (multiple peaks observed in the heavier portions of the gradient), as well as associated RNAs and proteins. We initially used Western blot analysis of the sedimentation of small ribosomal subunit protein S6 and large ribosomal subunit protein P0 to determine the identity of the peaks in the polysome profiles generated during fractionation (shown in Figures 6–8). Subsequent Western blot analysis was used to identify fractions containing canonical translation factors (shown using an antibody directed against eIF2α), PCBP2, and SRp20.

**Figure 7 ppat-1002127-g007:**
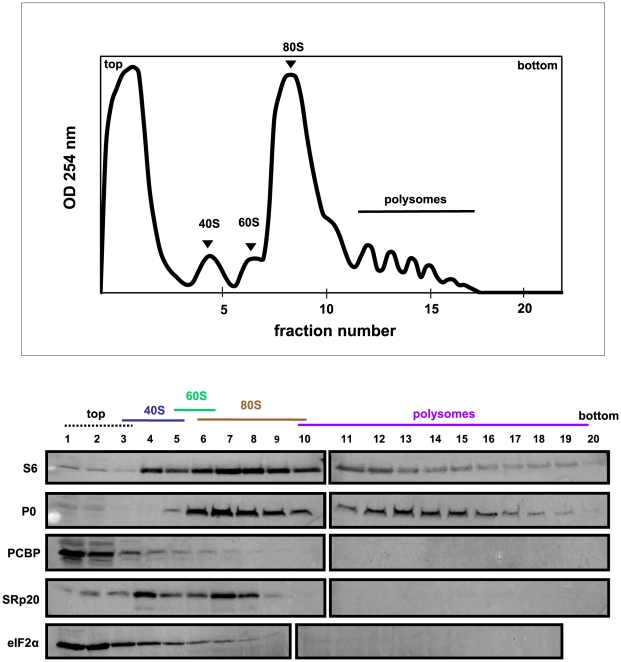
SRp20 and PCBP2 co-sedimentation with 40S ribosomal subunits in poliovirus-infected cells (2 hr post-infection). Extracts were generated from poliovirus-infected HeLa cells after 2 hours of infection and processed as described in the legend for Figure 6. Representative polysome profiles and Western blot analyses are shown.

**Figure 8:**
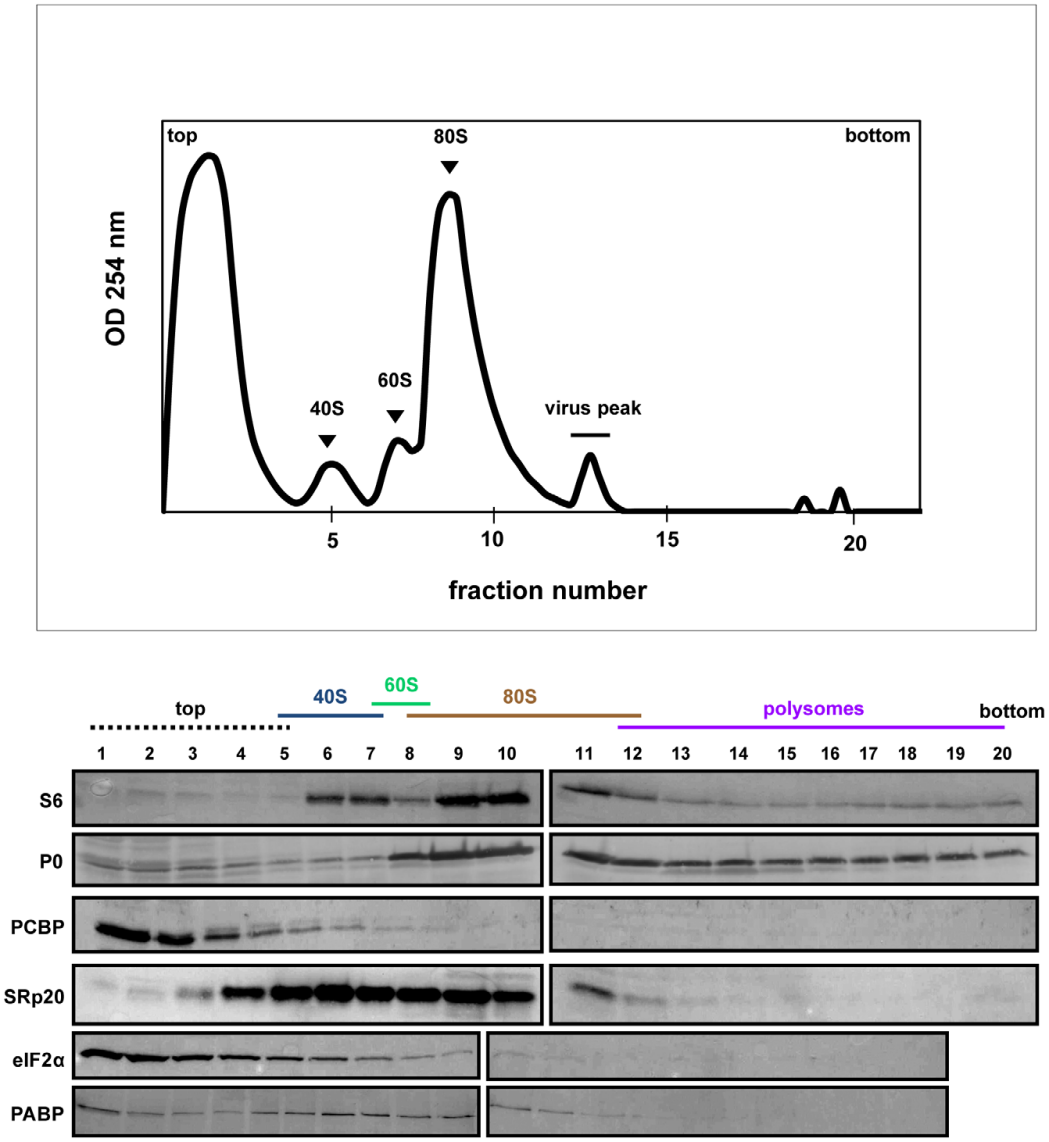
SRp20 and PCBP2 co-sedimentation with 40S ribosomal subunits in poliovirus-infected cells (5 hr post-infection). Extracts were generated from poliovirus-infected HeLa cells after 5 hours of infection and processed as described in the legend for Figure 6. Representative polysome profiles and Western blot analyses are shown. The sedimentation of virus (labeled ‘virus peak’ in the profile) was determined by Western blot analysis of fractions using an anti-VP2 polyclonal antibody that detects the poliovirus VP2 capsid protein (Holzberg, Nguyen, and Semler, unpublished).

In Figure 6, extracts from mock-infected cells consistently generated high levels of polysomes due to high levels of cap-dependent translation occurring in the cells. In extracts from mock-infected cells, PCBP2 was found mostly at the top of the gradient, as well as partially sedimenting in fractions containing 40S ribosomal subunits. SRp20 was found to sediment in 40S subunit-containing fractions, as well as 80S monosome-containing fractions. Neither PCBP2 nor SRp20 were found to co-sediment with polysomes, indicating these proteins do not remain associated with actively translating ribosomes. The sedimentation of PCBP2 somewhat resembles the sedimentation of eIF2α, a canonical translation initiation factor. Poly(A)-binding protein (PABP) was found to co-sediment with 80S monosomes and polysomes, displaying the importance of this protein in cap-dependent translation.

Figure 7 shows the polysome profile and Western blot analysis of fractions collected using extracts from poliovirus-infected cells at 2 hours post-infection. In this profile the polysome portion was reduced when compared to the profile for mock-infected cells. This would be expected, since at 2 hours post-infection much of the cap-dependent translation has been shut down. The polysomes observed here likely represent the remaining cap-dependent translation that was occurring, as well as poliovirus cap-independent translation. The Western blot analysis of these fractions was similar to that of the fractions collected from extracts from mock-infected cells; PCBP2 was found to sediment mainly at the top of the gradient as well as partially sedimenting in 40S fractions, while SRp20 was found in 40S and 80S fractions. Therefore, PCBP2 and SRp20 sediment in similar fractions when using extracts from mock- and poliovirus-infected cells.

The polysome profile and Western blot analysis of fractions collected using extracts from cells at 5 hours post-infection are shown in Figure 8. Interestingly, PABP is found to redistribute to the lighter portion of the gradient, and no longer co-sediments with polysomes at 5 hours post-infection. This observation is consistent with some published studies [13], [39] but not others [40]. There is also a lack of detectable polysomes at this time point late in infection, which is consistent with previously published work [41], [42], [43]. PCBP2 co-sediments partially with 40S ribosomal subunits, while SRp20 co-sediments with 40S subunits and 80S monosomes. Importantly, much more SRp20 appears in the fractions from cytoplasmic extract generated at 5 hours post-infection, which is consistent with our microscopy data indicating that much of the nuclear SRp20 re-localizes to the cytoplasm during poliovirus infection. Taken together, SRp20 and PCBP2 were found, at least in part, in fractions containing viral translation initiation complexes in both mock- and poliovirus-infected cells.

### SRp20 interacts with PCBP2 bound to poliovirus stem-loop IV

**Figure 9:**
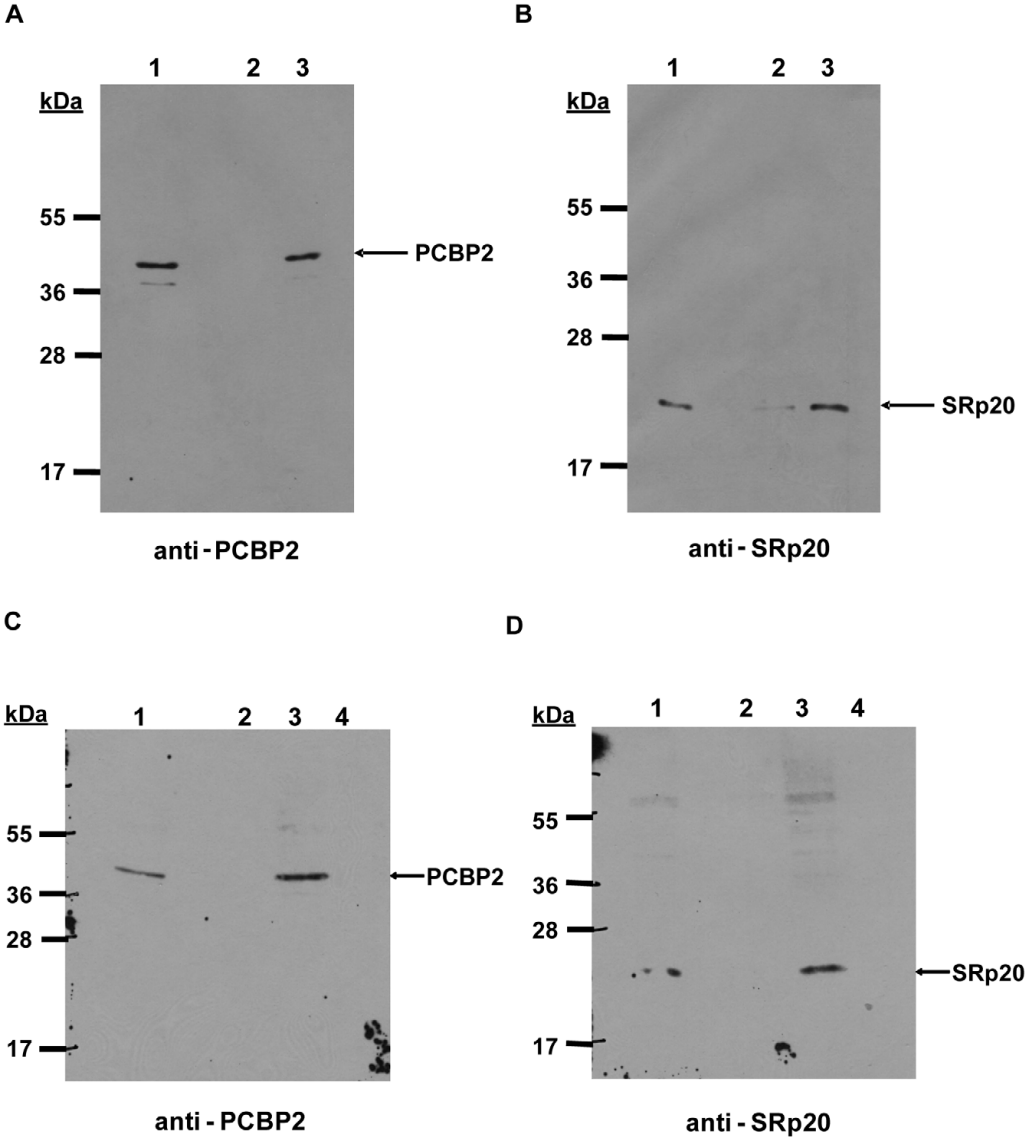
Interaction of PCBP2 and SRp20 on poliovirus stem-loop IV RNA. Poliovirus stem-loop IV RNA was transcribed and biotinylated with Biotin-CTP. The purified RNA (lane 3 in A and B) or tRNA alone (lane 2 in A and B) was incubated with streptavidin agarose, and the RNA-affinity matrix was subsequently incubated with extracts from poliovirus-infected cells (4 hours post-infection). Bound complexes were examined by Western blot analysis for the presence of PCBP2 and SRp20. Lane 1 in A and B, input extract (20% of experimental). The experiments were repeated exactly as described above, but included an additional experimental control. Extracts were incubated with tRNA alone (lane 2 in C and D), with biotinylated S-L IV RNA (lane 3 in C and D), or biotinylated S-L IV RNA and synthetic poly(rC) RNA (lane 4 in C and D) as a competitor for PCBP2 binding. Lane 1 in C and D, input extract (20% of experimental).

We next investigated whether SRp20 interacts with PCBP2 on poliovirus RNA. The interaction of PCBP2 with poliovirus RNA has been extensively studied in previous work [17], [44], [45]. Since SRp20 interacts with PCBP2 in extracts from poliovirus-infected HeLa cells, we wanted to determine whether this interaction occurs while PCBP2 is bound to the viral RNA. To address this question, we carried out RNA affinity assays using poliovirus stem-loop IV RNA and extracts from poliovirus-infected cells. Stem-loop IV RNA was transcribed and biotinylated using Biotin-CTP, the purified biotinylated RNA was incubated with extracts from poliovirus-infected cells, and bound complexes were isolated using streptavidin-agarose. Bound complexes were resolved and analyzed via Western blot. Figure 9 shows the representative results from four different RNA affinity assays. As expected, PCBP2 was associated with the biotinylated stem-loop IV RNA (Figure 9A, lane 3) but not with the negative control tRNA alone (Figure 9A, lane 2). Using this assay we were also able to determine that SRp20 is associated with PCBP2 bound to poliovirus stem-loop IV (Figure 9B, lane 3). Only background amounts of SRp20 were detected with tRNA alone (Figure 9B, lane 2). The RNA affinity experiments were repeated as described above, but with an additional control. When extracts were pre-incubated with synthetic poly(rC) RNA, previously shown to compete for PCBP2 binding [15], PCBP2 no longer interacted with the biotinylated poliovirus stem-loop IV RNA (Figure 9C, lane 4). In addition, SRp20 was no longer associated with S-L IV (Figure 9D, lane 4), suggesting that SRp20 is not associated with the viral RNA when PCBP2 is not bound. Thus, the interaction of SRp20 with PCBP2 appears to occur on viral RNA. Importantly, we have also utilized electrophoretic mobility shift assays to determine that SRp20 does not directly bind to poliovirus S-L IV (unpublished results).

### The subcellular localization of an RRM-deleted form of SRp20 is similar to the full-length SRp20 protein

Previous studies have outlined the requirement of the serine/arginine-rich (RS) domain of SRp20 for interaction with PCBP2 [18]. To better define the functional role of SRp20 in poliovirus IRES-mediated translation we generated a deleted form, SRp20ΔRRM, which contains a deletion of the entire RNA-recognition motif (RRM) but still possesses the RS domain. We hypothesized that an SRp20 protein lacking one of its functional domains would act as a dominant-negative protein when overexpressed in cells during poliovirus infection, effectively sequestering PCBP2 from functional viral translation complexes.

**Figure 10:**
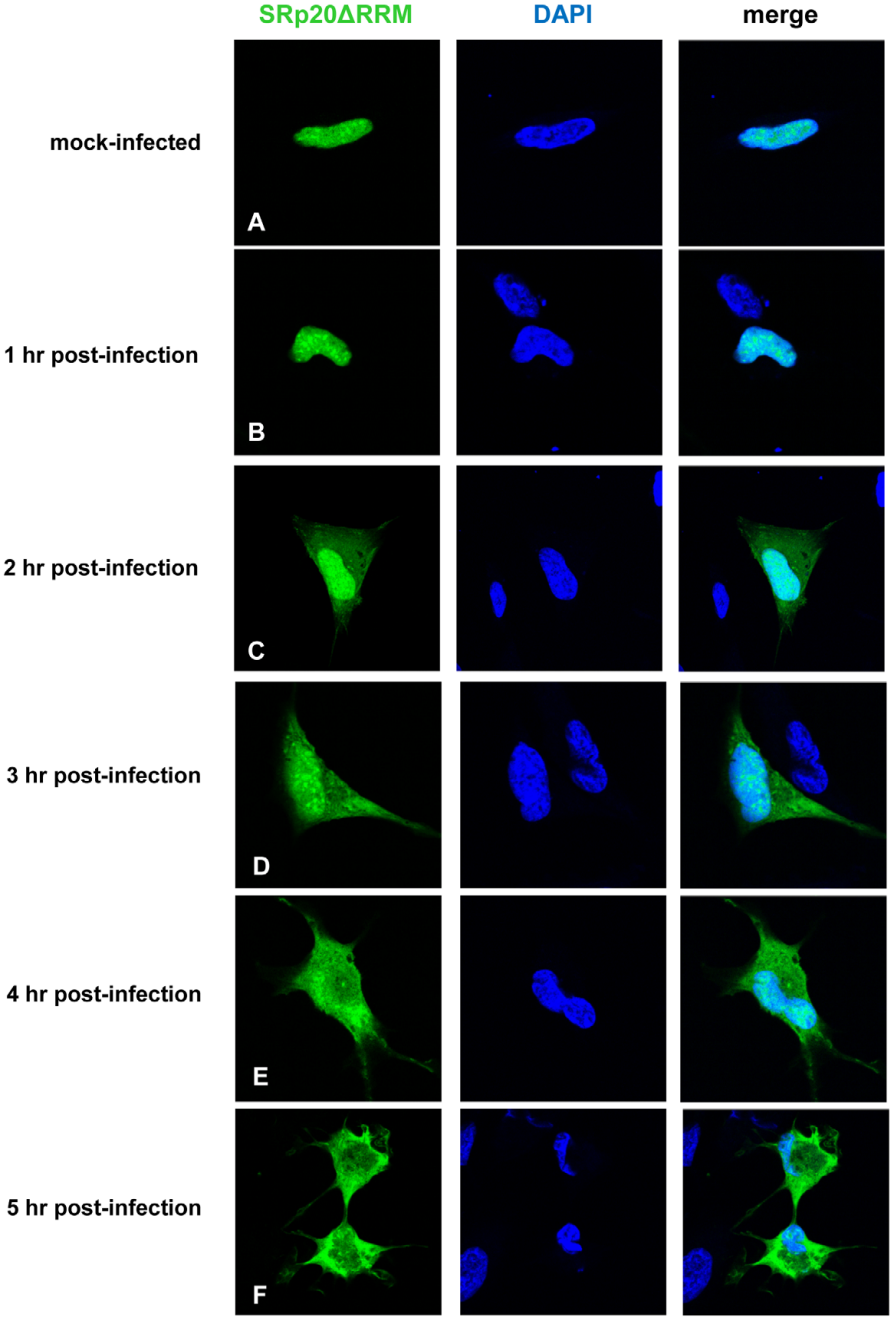
SRp20ΔRRM re-localization from the nucleus to the cytoplasm of SK-N-SH cells during poliovirus infection. Cells were transfected with GFP-SRp20ΔRRM and either mock-infected (A) or infected with poliovirus at an MOI of 25. Cells were fixed at specific times post-infection (1–5 hours, B–F) and imaged. SRp20ΔRRM localization was determined using confocal microscopy; nuclei were identified by DAPI staining.

To first determine the localization of this protein in the cell, the mutation was generated in the GFP-SRp20 clone; therefore, confocal microscopy could be employed to visualize its localization in mock- and poliovirus-infected cells. Since the RS domain acts as a nuclear localization signal for SR proteins [20], [46], [47], we predicted that the localization of the deleted form of the protein would be similar to wild type SRp20. This prediction was supported by studies on the Drosophila homologue of SRp20 (Rbp1); when the RRM domain was deleted from Rbp1 the protein localized like wild type Rbp1 in insect cells [48]. The results of our experiments in SK-N-SH cells are shown in Figure 10. Cells were mock-infected or infected with poliovirus, and at the indicated times cells were fixed and imaged to determine the localization of SRp20ΔRRM. Expression alone of the deleted form of SRp20 did not affect cell viability (data not shown). In mock-infected cells, SRp20ΔRRM was located predominantly in the nucleus, similar to the wild type SRp20 (see Figure 10A). The localization of SRp20ΔRRM in mock-infected cells did not change over the time course that was carried out (data not shown). During the course of poliovirus infection, the deleted form of SRp20 re-localized to the cytoplasm of the infected cell, which could be visualized beginning about 2 hours post-infection (Figure 10C). The localization of SRp20ΔRRM resembled the localization of wild type SRp20, as both proteins re-localized to the cytoplasm after poliovirus infection. SRp20ΔRRM accumulation in the cytoplasm of the cell increased over the course of infection (Figure 10D–F). Interestingly, SRp20ΔRRM appeared to re-localize to a somewhat greater extent at earlier times post-infection when compared to the wild type protein (compare Figures 2C and 10C). This observation was made in the majority of cells when comparing the localization of the full length and deleted forms of SRp20 during poliovirus infection.

### SRp20ΔRRM partially co-localizes with PCBP2 in the cytoplasm of poliovirus-infected SK-N-SH cells

**Figure 11:**
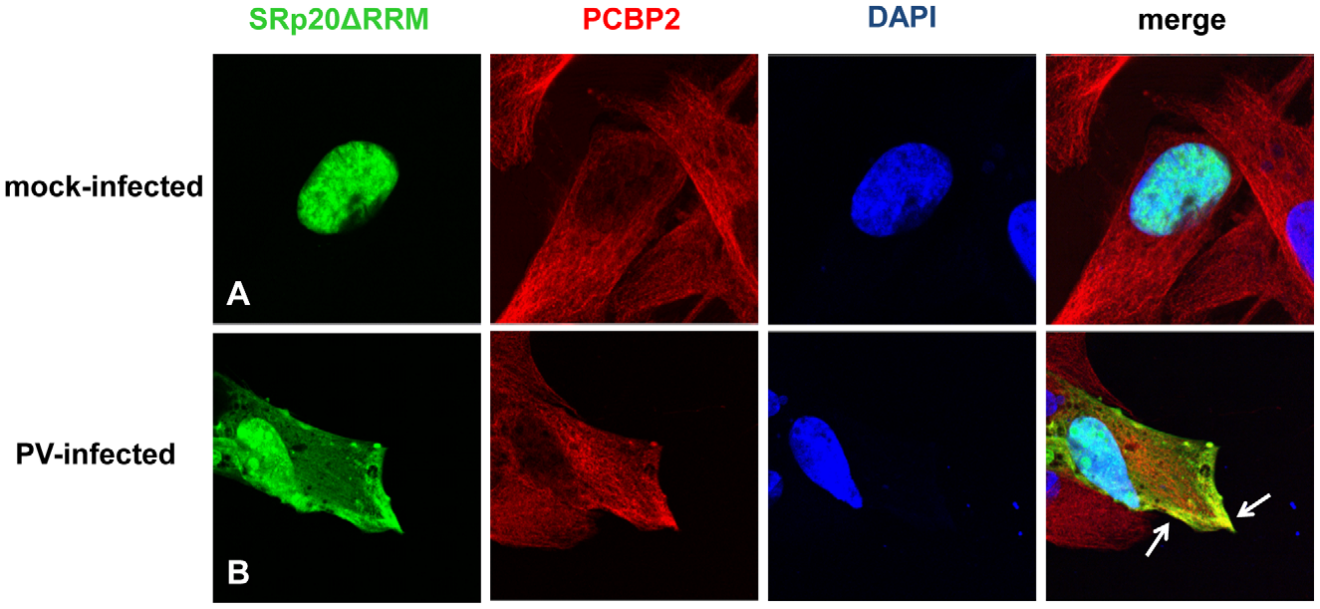
SRp20ΔRRM partial co-localization with PCBP2 in the cytoplasm of poliovirus-infected SK-N-SH cells. Cells were transfected with SRp20ΔRRM and mock-infected (A) or infected with poliovirus for 3 hours (B) at an MOI of 25. At 3 hours post-infection, cells were fixed and incubated with a PCBP2 monoclonal antibody, a secondary antibody conjugated to biotin, and streptavidin conjugated to Texas Red. Cells were examined for co-localization of PCBP2 and SRp20ΔRRM (shown in the merged image in yellow and highlighted by white arrows) in the cytoplasm of the cells using confocal microscopy; nuclei were identified by DAPI staining.

Because the deleted form of SRp20 contains the RS domain, we proposed that this protein would still interact with PCBP2, and therefore would be found in close proximity to PCBP2 in the cytoplasm of poliovirus-infected cells. To test this we carried out immunofluorescence experiments using a PCBP2 monoclonal antibody to investigate whether SRp20ΔRRM co-localized with PCBP2 during poliovirus infection. Utilizing confocal microscopy we again observed that SRp20ΔRRM was predominantly nuclear in the mock-infected cells, while PCBP2 was localized in both the nucleus and the cytoplasm (Figure 11A). We were able to determine that SRp20ΔRRM partially co-localizes with PCBP2 at 3 hours post-infection in the cytoplasm of the infected cell (see Figure 11B). This co-localization occurred in an area of the cytoplasm distinct from the nucleus, and was confirmed via z-stack analysis (data not shown). These experiments showed that SRp20ΔRRM was able to co-localize with PCBP2, which we predicted would occur since the deletion construct still contains the domain required for interaction with PCBP2. Interestingly, the co-localization appeared to occur in areas that likely do not contain active viral translation complexes (such as the cellular periphery), in contrast to what is seen for the full length SRp20. This observation would support our hypothesis that the deleted form of SRp20 may function as a dominant negative protein and sequester PCBP2 from active viral translation complexes, resulting in the formation of non-functional complexes found in areas of the cell where viral translation is likely not occurring.

### Expression of SRp20ΔRRM results in a decrease in poliovirus yield

**Figure 12:**
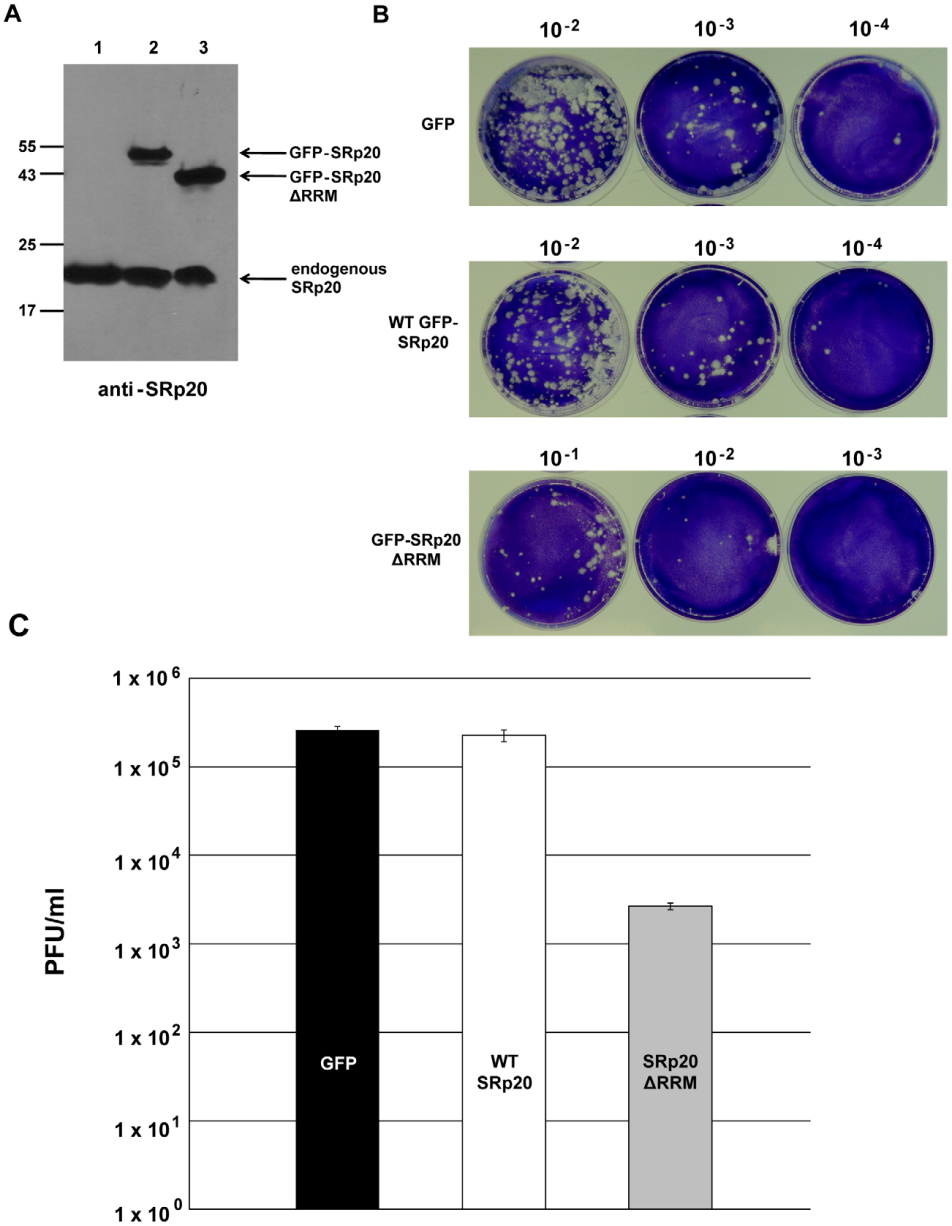
Effect of SRp20ΔRRM expression on poliovirus yield. Cells were co-transfected with poliovirus cDNA and a plasmid expressing either GFP alone, GFP-SRp20, or GFP-SRp20ΔRRM. Virus was harvested from the cells at 96 hours post-transfection and serially diluted. Dilutions of virus were used to carry out plaque assays (B), and whole cell extracts were also generated to determine the levels of expression of the SRp20 proteins by Western blot using an antibody against SRp20 (A). In (A) lane 1, cells transfected with the plasmid expressing GFP; lane 2, expressing GFP-SRp20; lane 3, expressing GFP-SRp20ΔRRM. Cells expressing the deletion mutant, GFP-SRp20ΔRRM, displayed a two-log decrease in poliovirus yield (C). The observed decrease (∼100 fold) was consistent across three separate experiments, although overall titers for the GFP control between experiments ranged from ∼10^5^ to ∼10^7^ (likely due to the variability of DNA transfection efficiency). Plaque assays to determine poliovirus titers were performed in triplicate.

If the deleted form of SRp20 functions as a dominant-negative protein, we would expect that its expression would result in a decrease in poliovirus yield. We predicted that SRp20ΔRRM, owing to its remaining RS domain, would interact with PCBP2 and effectively sequester PCBP2 from functional poliovirus translation complexes. If PCBP2 is sequestered away from functional viral translation complexes, this would hinder the progression of the infection and result in lower titers of virus produced. To test this we carried out DNA co-transfections, utilizing the GFP-tagged SRp20 or GFP-SRp20ΔRRM (or vector alone) and a recombinant plasmid harboring an infectious cDNA copy of the poliovirus (type 1) genome. This experimental design provided us the advantage of ensuring that every transfected cell would likely express both plasmids; therefore, virus would be produced under the influence of the expression of wild type SRp20 or the deleted form (or under the influence of expression from vector alone). This also provided an advantage in timing of expression, since the DNA plasmids should be expressed along a similar timeline. Importantly, however, the CMV promoter-driven expression of the full length and deleted forms of SRp20 would result in high levels of these proteins being generated well before any virus production from the poliovirus cDNA (see Figure 12A).

Cells were co-transfected with the poliovirus cDNA plasmid and either a plasmid expressing wild type SRp20 fused to GFP or SRp20ΔRRM fused to GFP (or GFP-expressing vector alone) and incubated at 37°C to allow for virus production. Whole cell extracts were also generated and probed with anti-SRp20 monoclonal antibody to ensure high levels of expression of both full length SRp20 and the deleted form (see Figure 12A). Following virus harvest and dilution, plaque assays were carried out to determine the effect of SRp20 or SRp20ΔRRM expression on virus growth. The representative results of these experiments are shown in Figure 12B and 12C. Expression of vector alone or of wild type SRp20 resulted in similar titers of poliovirus, while the expression of SRp20ΔRRM lowered the titers of virus produced by ∼100 fold. Thus, we conclude that expression of SRp20ΔRRM significantly reduced the levels of poliovirus produced, and that this protein may function as a dominant-negative in the cell.

## Discussion

Overall these results provide important new insights into the localization and functions of IRES trans-activating factors (ITAFs) during poliovirus infection. Splicing factor SRp20 was found to dramatically re-localize from the nucleus to the cytoplasm of the cell during poliovirus infection. Although other RNA binding proteins have been shown to re-localize to the cytoplasm of poliovirus-infected cells [35], [49], [50], [51], [52], [53], [54], this is the first time that a member of the SR family of proteins has been shown to display an altered localization and accumulation in the cytoplasm during poliovirus infection. The re-localization of SRp20 to the cytoplasm of the infected cell, where the virus life cycle occurs, is consistent with previous work from our lab showing the essential role of SRp20 in poliovirus IRES-mediated translation [18].

It is not yet clear whether SRp20 is more rapidly exported from the nucleus during poliovirus infection, whether its re-import from the cytoplasm is prevented during infection, if both processes are affected, and/or if other factors contribute to re-localization. Previous studies have demonstrated that SC35, another nuclear splicing factor, remains in the nucleus of poliovirus-infected cells at 3 hours post-infection, even though other import pathways are disrupted [35], [49]. Our findings for SRp20 localization during poliovirus infection are in stark contrast to that of SC35 at 3 hours post-infection; notably, SC35 is not a shuttling splicing factor. In the case of the SR family of proteins, these factors utilize Transportin-SR for nuclear import [46]. Because SC35 was found in the nucleus at 3 hours post-infection, this may indicate that the SR protein import pathway is intact during poliovirus infection because SC35, translated in the cytoplasm during infection, is still localized in the nucleus. It has also been demonstrated that some components of the nuclear pore complex are targeted for degradation during poliovirus infection, which likely affects multiple import and export pathways [35], [55]. This led to the conclusion that disruption in nuclear import is a factor in the cytoplasmic accumulation of some nuclear proteins (although possibly not SR proteins), while export may or may not be affected. Additional work has shown that nuclear export is also affected, and that an overall increase in the permeability of the nuclear envelope occurs during poliovirus infection, suggesting that protein trafficking is affected in a bidirectional way [56]. Interestingly, not all nuclear proteins re-localize to the cytoplasm of poliovirus-infected cells, and not all proteins that re-localize during infection necessarily do so with the same kinetics or to the same extent. For example, hnRNP C was found to re-localize to the cytoplasm of poliovirus-infected cells, but not to readily-detectable levels until 4 hours post-infection [35], [50]. Therefore, the specific cause of the re-localization of SRp20 during poliovirus infection remains to be determined, as does the potential role of other SR proteins (particularly other shuttling SR proteins) in poliovirus IRES-dependent translation.

We predicted we would be able to visualize PCBP2 and SRp20 co-localizing during infection, and thus in close proximity to each other in the cytoplasm of infected cells, because our lab has previously identified the interaction of these two proteins in extracts from poliovirus-infected cells via co-immunoprecipitation assays [18]. Our imaging data of the co-localization of PCBP2 and SRp20 corroborates our previous *in vitro* interaction data using recombinant proteins and GST pull down assays, and illustrates the close proximity of PCBP2 and SRp20 in the cytoplasm of intact cells during poliovirus infection.

Using sucrose gradient fractionation of extracts from mock- or poliovirus-infected cells, we detected both PCBP2 and SRp20 in fractions containing 40S ribosomal subunits; SRp20 could also be detected in fractions containing 80S monosomes. The significance of SRp20 co-sedimentation with 80S monosomes is not yet known, although other SR proteins have been shown to co-sediment with 80S monosomes or polysomes for cap-dependent translation [24], [25], [26]. The sedimentation of PCBP2 resembles, in part, the sedimentation of a canonical translation initiation factor eIF2α. Importantly, PABP co-sedimentation with polysomes in extracts from mock-infected cells is consistent with its function in cap-dependent translation, and validates our assay. The co-sedimentation of SRp20 and PCBP2 with 40S ribosomal subunits in extracts from poliovirus-infected cells is consistent with these proteins functioning in translation initiation complex formation for poliovirus IRES-mediated translation. It is possible that these proteins are present in viral translation initiation complexes, and are released from the assembled ribosome at or just after 60S subunit-joining. The release of canonical translation factors from cap-dependent translation initiation complexes is known to occur, and these factors are recycled for subsequent rounds of initiation [57]. SRp20 and PCBP2 are also found to co-sediment with 40S subunits in extracts from mock-infected cells. This would suggest that the virus is taking advantage of a yet undiscovered mechanism already present in the cells, which then functions to initiate translation of the viral RNA. Further fractionation and mass spectrometry analysis of 40S gradient fractions will be required to identify other factors involved in the formation of initiation complexes for poliovirus translation, including those capable of interacting with PCBP2 and/or SRp20.

RNA affinity assays showed that SRp20 is associated with PCBP2 bound to poliovirus stem-loop IV in the 5′ NCR of genomic RNA. Previous work had defined the direct binding of PCBP2 to poliovirus stem-loop IV [44], [58]; importantly, SRp20 does not appear to bind stem-loop IV directly (unpublished results). This suggests that SRp20 is associated with the viral RNA via its interaction with PCBP2. This interaction may then function to recruit the translation complex to the viral RNA either directly or indirectly, through other protein-protein or protein-RNA associations. Future studies will focus on identifying other factors that affinity-purify with poliovirus RNA and/or interact with SRp20 directly.

The RS domain of SRp20 can be highly phosphorylated, and its phosphorylation is important for its functions in the cell (e.g., splicing and nuclear export, for review, see [23]). The phosphorylation state of SRp20 when interacting with PCBP2 bound to poliovirus stem-loop IV has not been completely defined. Western blot analysis using an antibody that recognizes only the phosphorylated forms of SR proteins (pan-SR hybridoma supernatant mAb104, a gift from Dr. Roz Sandri-Goldin) suggested that little of the SRp20 that co-purified with PCBP2 and poliovirus stem-loop IV was phosphorylated (data not shown). In addition, co-immunoprecipitation assays after alkaline phosphatase treatment (or mock-treatment) of extracts from poliovirus-infected cells to dephosphorylate proteins in the extracts indicated that only unphosphorylated SRp20 interacts with PCBP2 in these extracts (data not shown). These preliminary results suggest that the unphosphorylated form of SRp20 is important for poliovirus translation, although additional analyses will need to be carried out to confirm this prediction.

Our experiments with a deleted form of SRp20 (SRp20ΔRRM) demonstrated that its localization was similar to that of the wild type protein: it was found mainly in the nucleus of mock-infected cells and re-localized to the cytoplasm of poliovirus-infected cells. Our findings are consistent with published data for the Drosophila homologue of SRp20 (Rbp1), that even when lacking the RRM domain still localized like the wild type protein in insect cells [48]. The nuclear localization of SRp20ΔRRM in mock-infected cells was expected since the RS domain acts as a nuclear localization signal for SR proteins. It was initially predicted that the deleted form of SRp20 would in fact re-localize to the cytoplasm during poliovirus-infection since the mutated protein still contains the portion of the protein required for TAP interaction and export [30], although we do not yet know the mechanism by which SRp20 re-distributes to the cytoplasm of the cells during poliovirus infection.

Co-localization of PCBP2 and SRp20ΔRRM suggests that these two proteins are in close proximity in the cytoplasm of the cell during infection. SRp20ΔRRM lacks one of its two defined functional domains, thus we predicted that this truncated protein would still interact with PCBP2 (since it contains the RS domain required for PCBP2 interaction) but would result in a non-functional interaction for poliovirus translation. Indeed, expression of SRp20ΔRRM resulted in an approximate two-log decrease in virus yield for poliovirus when compared to expression of wild type SRp20 or vector alone. These results and the co-localization data suggest that SRp20ΔRRM can still interact with PCBP2, but in a non-functional complex for poliovirus infection. We propose that SRp20ΔRRM acts as a dominant negative protein and effectively sequesters PCBP2 from functional translation complexes, leading to a decrease in virus yield. We do not yet know the mechanism of the dominant negative function of SRp20ΔRRM, although ongoing studies will elucidate whether SRp20ΔRRM can interact with PCBP2 bound to the viral RNA, determine the kinetics of the poliovirus growth defect observed with expression of SRp20ΔRRM, and investigate the specific effect of SRp20ΔRRM expression on viral translation.

Overall these studies have provided new evidence for the importance of PCBP2-SRp20 interaction in intact cells during poliovirus infection and suggest a model by which PCBP2, bound to the viral RNA, interacts with SRp20 and functions to recruit the translation initiation complex to the viral RNA. The localization of SRp20 changes dramatically during poliovirus infection, and its accumulation in the cytoplasm would allow for its continued availability for poliovirus translation. In addition, when SRp20 lacks one of its two defined functional domains its expression results in a decrease in virus yield for poliovirus. This finding further underscores the significance of SRp20 for poliovirus infection. On a broader scale, this work provides insights into potential mechanisms of ribosome recruitment for picornavirus IRES-mediated translation initiation, and how non-canonical factors play a role in bridging the translation machinery to the RNA.

## Supporting Information

Video S1 **Subcellular 3-D analysis of PCBP2-SRp20 co-localization.** Z-stack analysis was performed for some of the images collected for the co-localization studies (data not shown). Z-stacks were rendered into three dimensions using the Volocity Image Analysis Software (PerkinElmer) to generate QuickTime movies of the cells in rotation to further investigate the subcellular location of co-localization in the cytoplasm. GFP-SRp20 is displayed in green, PCBP2 is shown in red, and areas of yellow in the cytoplasm indicate co-localization.
(MOV)
